# Evaluation Method for Creep Damage of P92 Steel Based on Magnetic Barkhausen Noise and Magnetoacoustic Emission

**DOI:** 10.3390/s26061909

**Published:** 2026-03-18

**Authors:** Ziyi Huang, Wuliang Yin, Xiaochu Pang, Xinnan Zheng, Xufei Liu, Lisha Peng

**Affiliations:** 1 Department of Electrical and Electronic Engineering, The University of Manchester, Manchester M13 9PL, UK; ziyi.huang-7@postgrad.manchester.ac.uk (Z.H.); xiaochu.pang@postgrad.manchester.ac.uk (X.P.); xinnan.zheng@postgrad.manchester.ac.uk (X.Z.); 2 Department of Electrical Engineering, Tsinghua University, Beijing 100084, China; liuxf22@mails.tsinghua.edu.cn (X.L.); penglisha@mail.tsinghua.edu.cn (L.P.)

**Keywords:** creep damage evaluation, magnetoacoustic emission, magnetic Barkhausen noise, principal component analysis

## Abstract

The application of ultra-supercritical power plant boilers is becoming increasingly widespread. P92 steel, as a typical material used for boiler main steam pipes, plays a critical role in unit safety, making the detection of its creep damage highly significant. However, existing conventional non-destructive testing methods are difficult to effectively detect creep damage. To address this issue, a magnetoacoustic emission (MAE)–magnetic Barkhausen noise (MBN) composite measurement system is developed, which is adapted to 20 Hz and 0.3 A sine wave excitation to trigger the synchronous pickup of MBN and MAE signals of P92 steel. After collecting signals with different creep life ratios (0%~100%) under working conditions of 650 °C and 100 MPa, time-domain (absolute mean, peak value, etc.) and frequency-domain (bandwidth) features are extracted. In response to the non-monotonicity between the magnetoacoustic features and the creep damage grade, principal component analysis (PCA) is introduced to reduce dimensionality. Different creep levels of samples in the two-dimensional principal component space are presented as clear gradient clustering, achieving the accurate differentiation of creep stages. Research has shown that the MAE-MBN composite system combined with PCA can effectively characterize the creep damage of P92 steel, providing a novel non-destructive detection path for the in-service life assessment of power plant components.

## 1. Introduction

Ultra-supercritical power plants play a crucial role in global energy supply, and P92 steel has been widely adopted for critical components such as main steam pipes due to its superior high-temperature endurance and thermal conductivity. However, prolonged exposure to high-temperature and high-pressure environments subjects the material to inevitable creep damage under the combined influence of stress, temperature, and time. This creep damage manifests as microstructural evolution such as dislocation proliferation, Laves phase coarsening, and the accumulation of grain boundary pores, ultimately threatening the safe and efficient operation of power plants [1]. Therefore, achieving the early and accurate detection of creep damage is of great significance for preventing catastrophic failures and optimizing maintenance strategies.

However, the conventional non-destructive testing methods, such as magnetic flux leakage, ultrasonic, and eddy current testing, are limited to the detection of macroscopic defects at the submillimeter level or above, and are generally ineffective for detecting creep damage at the microscale. To address this limitation, magnetic Barkhausen noise (MBN) and magnetoacoustic emission (MAE) are utilized in this work for creep damage detection. Multiple research teams around the world have conducted extensive explorations on the optimization and application of two types of technologies. In the field of MBN technology, Baldev et al. discussed the feasibility of using the magnetic Barkhausen noise (MBN) detection method to characterize microstructure, deformation, fatigue, and creep damage for different steels. Through theoretical modeling, they detected and identified creep damage at various stages in 9Cr-1Mo steel, laying a theoretical foundation for early creep damage detection [2]. Mierczak et al. presented a magnetic Barkhausen emission method for detecting surface stress in steels, utilizing a linear calibration curve between the reciprocal of peak emission amplitude and stress for quantitative measurement [3]. Deng et al. demonstrated that magnetic Barkhausen noise parameters strongly depend on material structure characteristics like carbon content and hardness, which is explained by domain wall nucleation and propagation mechanisms, advancing its use in non-destructive evaluation [4]. Results showed that MBN parameters strongly depend on material structure, with the carbon content exhibiting a non-monotonic influence by increasing MBN up to a peak at 0.55 wt% before decreasing.

The research progress of MAE technology mainly focuses on its detection sensitivity to early structural damage and the analysis of signal characteristics. Shen et al. reported that MAE shows promise for early fatigue damage evaluation, as its RMS value decreases with accumulated fatigue cycles and provides an indicator for the onset of macrocrack propagation, potentially serving as a non-destructive alternative to conventional methods [5]. Riaz et al. noticed that non-contact coil excitation provides stable MAE baselines, whereas contact-based electromagnet excitation offers higher sensitivity but with greater variability, underlining key considerations for industrial NDT application [6]. However, the MAE technology faces challenges in distinguishing signals from background noise at lower frequencies. Additionally, its low signal-to-noise ratio means that disturbances during signal processing can lead to measurement errors [7,8].

The limitations of single-technique detection have promoted the development of MBN-MAE composite detection technology. Rocío et al. proposed that the complementary non-destructive techniques of MBN and MAE, which arise from domain wall motion and associated stress waves, respectively, can be utilized to characterize the microstructure of various stainless steels under different magnetic field orientations [9]. Astudillo et al. combined MBN, MAE, and microstructural analysis and found that the magnetic anisotropy in forged nuclear steel reveals a plastic flow direction inconsistent with the documented forging axis, linking magnetic signatures to underlying texture [10]. Skalskyi et al. proposed that MAE excited by the magnetic field via the Barkhausen effect can circumvent the loading limitation of traditional AE and serve as an effective method for detecting material degradation, such as hydrogen-induced damage, while also reviewing its underlying theoretical models [11]. Astudillo et al. concluded that MBN and MAE each serve as an effective tool for studying the microstructure and plastic deformation of ferromagnetic materials, and that integrating both methods can provide a basis for fatigue life prediction [12].

Recent studies further emphasized that advanced magnetic signal analysis combined with multi-parameter feature extraction or statistical approaches can significantly enhance the sensitivity of magnetic techniques to early-stage degradation, providing a more reliable basis for damage assessment in high-temperature steels [13].

Despite significant breakthroughs in existing research, the quantitative correlation model between signal characteristics and creep damage degree has not been unified, making it difficult to achieve the accurate division of creep stages [14,15]. Therefore, this paper focuses on studying the variation patterns of magnetic Barkhausen noise (MBN) and magnetoacoustic emission (MAE) signals during the creep evolution of P92 steel. This research aims to provide a reference for the combined magnetoacoustic detection and assessment of creep damage, thereby facilitating the safe operation and maintenance of ultra-supercritical power plant equipment.

## 2. Principle of MBN and MAE Detection for Creep Damage

### 2.1. Mechanisms of MBN and MAE Signals

#### 2.1.1. Core Physical Mechanism of MBN Signal

MBN is an electrical pulse signal generated by the discontinuous jumping motion of magnetic domain walls in ferromagnetic materials during dynamic magnetization. Its essence is directly related to the evolution of the internal magnetic domain structure of the material [16]. The magnetic domains formed by the spontaneous magnetization of ferromagnetic materials have different magnetization directions, and adjacent magnetic domains are separated by domain walls. Under normal conditions, the arrangement of magnetic domains is in energy balance. When an external alternating magnetic field acts on a material, the magnetic domains rotate along the easy magnetization direction, while the domain walls overcome internal resistance and undergo displacement.

During the high-temperature creep process of P92 steel, microstructural changes such as dislocation proliferation, Laves phase coarsening, and grain boundary voids can form “pinning points” for magnetic domain wall motion [17]. These pinning points hinder the continuous movement of magnetic domain walls. When the magnetic field strength is sufficient to break through the pinning point constraint, irreversible jumping motion occurs, causing a sudden change in the internal magnetic flux of the material, which in turn induces a series of irregular pulse signals, namely MBN signals, in the surrounding detection coils [18]. The more severe the creep damage, the more internal micro defects, and the more significant the magnetic domain wall pinning effect; the characteristic parameters of MBN signal also show regular changes accordingly. Research has confirmed that there is a strong correlation between the changes in MBN signal during the service life of P92 steel and the evolution of precipitation phases, as well as the degradation of mechanical properties, which can directly reflect the aging state of the material.

#### 2.1.2. Core Physical Mechanism of MAE Signal

MAE is based on the magnetostriction effect of ferromagnetic materials, which means that the material undergoes small changes in volume or shape during magnetization [19]. During the high-temperature creep process of P92 steel, internal damage such as micro stress concentration, microcrack initiation and propagation occur, which disrupt the stress balance inside the material and cause the rearrangement of the magnetic domain structure.

When an external magnetic field is applied, the reorientation of magnetic domains can cause the local magnetostrictive deformation of the material, which is released in the form of elastic waves, forming the MAE signals [20]. Unlike the MBN signals, the generation of MAE signals is closely related to the 90° rotation of magnetic domain walls, and is more sensitive to stress concentration and microcracks inside the material. In the early stage of creep, the MAE signal is mainly caused by a magnetic domain rearrangement due to micro stress concentration. After entering the middle and late stages, the propagation of microcracks will cause significant changes in the amplitude and velocity of MAE signals, providing a basis for the division of damage stages [21]. Under high-temperature conditions, the propagation characteristics of MAE signals are directly related to the degradation of material elastic modulus, which can indirectly reflect the decrease in mechanical properties caused by creep.

### 2.2. Key Characteristic Parameters and Physical Meanings of MBN and MAE Signals

The characteristic parameters of MBN and MAE signals are the core carriers reflecting the creep damage state. Common parameters include absolute mean value, peak value, ringing number and bandwidth. Each parameter has a clear physical meaning.

Absolute mean value: It is the average intensity of irreversible jumping of magnetic domain walls per unit time, and it increases as the stress in the material increases.

Peak value: It is the difference between the positive peak of the signal and ground, which is significantly sensitive to the changes in magnetic domain wall constraints caused by the coarsening of the precipitate phase.

Ringing number: It is the number of pulses whose amplitude exceeds the set threshold, reflecting the frequency of magnetic domain flipping, but it is greatly affected by signal randomness and has high dispersion [22].

Bandwidth: It is the width of the signal frequency that is related to the magnetic field change rate and magnetostriction coefficient.

These characteristic parameters are quantitatively correlated with the creep damage state of P92 steel. By establishing a mapping relationship between characteristic parameters and the degree of creep damage, the non-destructive evaluation of the creep state of P92 steel can be achieved.

### 2.3. Collaborative Complementary Mechanism for MBN and MAE Bimodal Detection

The detection depth of MBN and MAE technologies is determined by the skin effect of the excitation magnetic field, which is closely related to the conductivity, permeability, and excitation frequency of the material. In this experiment, under the low-frequency excitation condition of 20 Hz, the skin depth is roughly in the order of millimeter. The MBN technology is highly sensitive to microstructural changes on the surface of materials (<1 mm) and can accurately capture early damages such as surface dislocations and coarsening of precipitated phases; the MAE technology relies on the propagation characteristics of elastic waves, which can effectively cover the near surface (≤10 mm) area, and it has better detection capabilities for damage such as sub-surface stress concentration and micropore aggregation. The complementarity between the two in terms of detection depth has achieved full-layer damage monitoring from the surface to the near surface of P92 steel specimens, improving the comprehensiveness of damage detection. At the same time, MAE can only effectively detect defects within 10 mm, as low-frequency elastic waves propagate in P92 steel and their energy rapidly decays beyond this depth, resulting in a significant decrease in detection sensitivity.

The MBN and MAE technologies have significant synergistic complementarity in the creep detection of P92 steel, which can achieve full-cycle damage coverage.

Complementary detection objects: The MBN technology mainly responds to the 180° jump of magnetic domain walls and is highly sensitive to the microstructure evolution in the early stage of creep (such as dislocation proliferation and coarsening of precipitated phases), which can achieve the early warning of damage. The MAE technology focuses on the 90° rotation of magnetic domain walls, which is more sensitive to micro stress concentration and microcrack propagation in the middle and late stages, and excels in damage localization and quantitative evaluation.

Complementary feature information: The energy parameters of MBN signals (such as RMS and envelope peak) reflect the cumulative degree of damage, while the wave velocity and spectral parameters of MAE signals reflect the spatial distribution and development trend of damage. The combination of the two can construct a multidimensional and comprehensive damage assessment system.

This collaborative mechanism enables dual-mode detection technology to cover the full creep cycle of P92 steel from early micro deterioration to late macro failure, significantly improving the accuracy and reliability of detection, and providing technical support for the safe operation and maintenance of key equipment such as ultra-supercritical power plant boilers.

## 3. Experimental System Construction

In order to achieve synchronous and stable acquisition of the MBN and MAE signals of P92 steel plate specimens and fully utilize the complementary advantages of the two technologies in detection depth—MBN’s high sensitivity to surface (<1 mm) damage and MAE’s effective coverage of near-surface (up to 10 mm) defects—a MBN and MAE composite detection system is built, and integrated measurement and control software is developed. The hardware configuration and software functions of the system were customized around the core experimental requirements to ensure the accuracy of signal acquisition and the efficiency of data analysis.

### 3.1. Preparation of Experimental Samples

**Figure 1:**
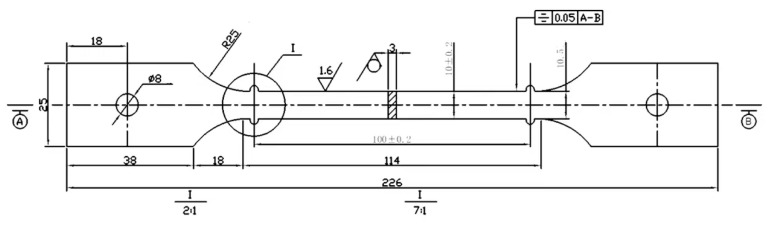
Design diagram of high-temperature creep specimen.

To investigate the creep damage evolution mechanism of ferromagnetic high-temperature alloy P92, the P92 experimental specimen shown in Figure 1 was prepared in accordance with the Chinese national standard [23]. The specimen is a dumbbell-shaped structure with the following key dimensional parameters: a total length of 226 mm, a gauge length of 100 ± 0.2 mm, a transition arc radius of R25, a width of 38 mm and a thickness of 10 mm at both ends of the clamping section, and an installation hole with a diameter of 8 mm. The overall dimensional accuracy meets the requirements for clamping and force uniformity in creep testing.

This experiment conducted creep tests on P92 steel at 650 °C and 100 Mpa. In creep testing, the specimens are conducted under nitrogen protection. The temperature of the specimen gauge section is monitored in real time by thermocouples, with temperature fluctuations controlled within ±2 °C. The temperature rise rate is 8 °C/min. The stress is loaded to 100 MPa within 10 min. The creep life was determined by the fracture time of the specimen, which was 1117 h. Based on this life value, creep interruption nodes of 10%, 20%… 90% were set. The sensing module of the MBN and MAE composite detection system was fixed on the surface of the gauge section 50 mm away from the installation hole of the specimen, ensuring that the detection area was in the uniform stress section of the specimen and avoiding interference from stress concentration on the signal.

Independently prepared P92 steel samples which is manufactured by Nantong University, Nantong, China were used for different creep life stages (0%, 10%, 20%, …, 100%) in this experiment. Three parallel samples were set for each life stage, for a total of 33 samples. Six sets of magnetoacoustic signal data were collected for each sample, resulting in a dataset of 18 sets per life stage. When collecting data for each sample, the sensor is located at the same position on the sample. The experiment ensured consistency between different samples by strictly controlling the sample preparation process and experimental parameters (temperature, stress accuracy), providing a foundation for the statistical robustness and reproducibility of experimental results.

### 3.2. Overall System Structure and Working Logic

**Figure 2:**
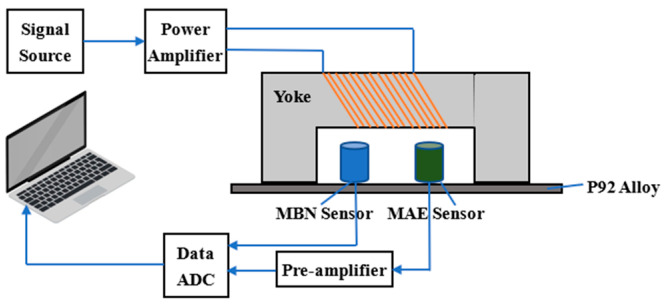
The overall framework of the MBN and MAE composite detection system.

The overall architecture of the MBN and MAE composite detection system is shown in Figure 2, with excitation magnetic field loading, signal pickup and data processing as the core process.

It mainly consists of a signal source module, a power amplification module, an excitation yoke module, a sensing and detection module, a preamplifier module, a data acquisition module and an upper computer module.

The excitation signal output by the signal source module is amplified by a power amplifier, which drives the magnetizing yoke to generate an alternating magnetic field and acts on the P92 alloy sample. The MBN and MAE signals generated by the sample under magnetic field excitation are synchronously picked up by the corresponding sensors and transmitted to the preamplifier for signal enhancement. The processed signal is converted into a digital signal by the data acquisition module and finally uploaded to the upper computer for filtering, storage and analysis. The detection system utilizes the magnetization signal to trigger dual-channel data acquisition for MBN and MAE signals.

### 3.3. Customized Design of Excitation Module

The excitation module is the core of the system to generate a stable alternating magnetic field, corresponding to the signal source, power amplifier and magnetizing yoke in Figure 2. Its parameters have been optimized through multiple experiments to adapt to the magnetic characteristics of P92 steel.

The signal source module uses a programmable signal generator, which can output various waveforms such as sine wave, triangular wave, square wave, etc. In the experiment, the default output frequency is 20 Hz and the peak-to-peak value is 8 V sine signal—this frequency matches the characteristic frequency of P92 steel magnetic domain wall motion, which can effectively produce a magnetic domain rearrangement and avoid additional magnetic loss caused by high-frequency magnetic fields. The output signal of the signal source is amplified by a TDA2030 power amplifier to 0.3 A current, ensuring that the magnetizing yoke can generate a sufficiently strong magnetic field.

The magnetizing yoke adopts a U-shaped structure, which is suitable for the surface magnetic field loading requirements of P92 steel plate specimens. The magnetizing yoke is made of stacked silicon steel sheets with a thickness of 0.35 mm, and its middle is wrapped with a 600-turn enameled wire coil with a diameter of 0.25 mm.

### 3.4. MBN and MAE Signal Pickup and Conditioning Module

This module corresponds to the MBN sensor, MAE sensor and Preamplifier in Figure 2, responsible for synchronously picking up and enhancing the MBN and MAE response signals of the sample. The selection and installation of core components are designed around optimizing signal accuracy.

Accurate positioning of sensors based on 3D printing fixtures: MBN sensors and MAE sensors are fixed on the surface of P92 samples in a coplanar manner, with a spacing controlled within 15 mm (to avoid signal crosstalk), and both are located near the magnetic pole of the magnetizing yoke.

In terms of installation process, the MAE sensor (Qingcheng G150 split single-end type, manufactured in Guangzhou, China) is uniformly coated with a high-temperature resistant coupling agent on the surface of the sample to eliminate air gaps and improve the efficiency of elastic wave transmission.

#### 3.4.1. Sensor Layout and Installation

The MBN sensor (manganese zinc ferrite core 1000-turn coil) is directly attached to the surface of the sample, which detects the normal component of the magnetic field variety.

#### 3.4.2. Preamplifier Configuration

The preamplifier used is SAEPA2 from Qingcheng Acoustic Emission Research (Guangzhou) Co., Ltd (Guangzhou, China). Its core function is to enhance weak MAE signals. The gain of the amplifier was set to 40 dB in the experiments. The frequency band was switched within the range of 100–200 kHz to achieve targeted filtering and denoising.

### 3.5. Data ADC and Analysis Software

The data acquisition module uses the ART USB2872 acquisition card (manufactured by Art Beijing Science and Technology Development Co., Ltd., Beijing, China), with a sampling rate of up to 1 MHz and a resolution of 16 bits. It supports two-channel synchronous acquisition of MBN and MAE, with a sampling depth of 1 M samples/channel, meeting the large data storage requirements for long-term creep damage detection experiments. The acquisition card is connected to the upper computer through a USB 3.0 interface, with a data transmission rate of 5 Gbps, ensuring a real-time signal without any delay.

The analysis software on the upper computer was developed based on the LabVIEW platform and integrates three core functions:

Magnetizing control function: Set the waveform, frequency and amplitude of the signal source, display the magnetizing current curve in real time, and support dynamic parameter adjustment.

Signal acquisition and conditioning function: Collect multi-channel signals at a sampling rate of 1 MHz, with a built-in Butterworth bandpass filter for noise reduction, while displaying real-time time-domain waveforms and spectral distributions.

Data storage function: Store the original signal and extracted feature parameters (such as MBN root mean square value, MAE amplitude) in the TDMS format. A single file supports the continuous recording of experimental data for more than 10 h and is compatible with software such as Microsoft 365 Excel and MATLAB 2025b.

**Figure 3:**
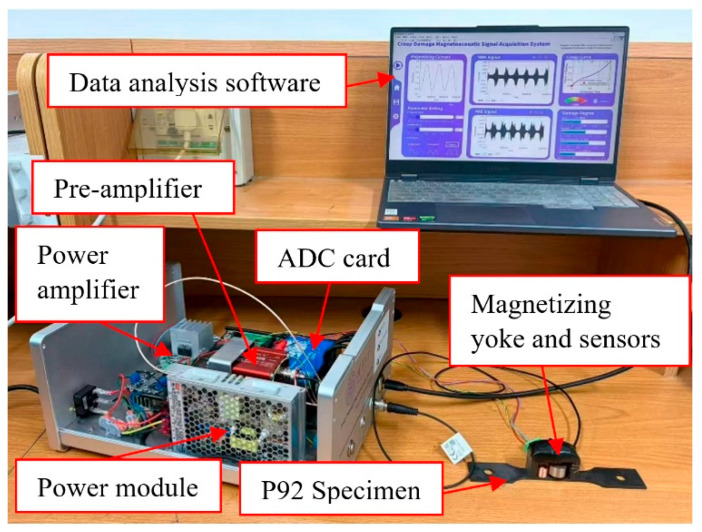
The photo of the MBN and MAE composite detection system.

The entire experimental system (Figure 3) has constructed a complete technical chain from magnetic field excitation to signal acquisition, processing and storage through precise matching of hardware units and integrated design of software, achieving multi-signal joint detection of creep damage on the surface and near surface of P92 steel plate specimens. It provides a stable and reliable experimental platform for the in-depth analysis of the evolution law of MBN and MAE signals with the creep process.

### 3.6. System Stability and Repeatability Verification

The results of signal feature repeatability, long-term drift characteristics, and sensor relocation error showed that the system performance was stable and the signal feature variance was at a low level.

Signal feature repeatability: For the same non-creep P92 steel sample, 20 sets of MBN and MAE signals were continuously collected under the same excitation and detection parameters, and core feature parameters such as absolute mean and peak value were extracted to calculate their coefficient of variation (CV). The results showed that the coefficient of variation in all feature parameters was less than 3%, indicating good signal acquisition repeatability of the system.

Long-term drift characteristics: The sample was subjected to 2 h of continuous signal acquisition to monitor the trend of characteristic parameter changes. The results showed that the drift of characteristic parameters was within ±2% range, with no obvious drift phenomenon, meeting the requirements of long-term online monitoring.

Sensor relocation error: After disassembling and reinstalling the sensor to the same detection area of the sample, 10 sets of signals were collected repeatedly. The deviation rate of the characteristic parameters was less than 4%, indicating that sensor relocation has a small impact on the detection results.

The above experimental results demonstrate that the MAE-MBN composite detection system developed in this study has good stability and repeatability, and the variance of its signal characteristics is at a low level, which can meet the detection needs of experiments.

## 4. Data Analysis

The quality of MBN and MAE signals directly determines the reliability of creep damage feature extraction. This section focuses on the selection of acquisition parameters, filtering preprocessing and waveform features of MBN and MAE signals in experiments.

### 4.1. Creep Curve and Metallographic Analysis Results

**Figure 4:**
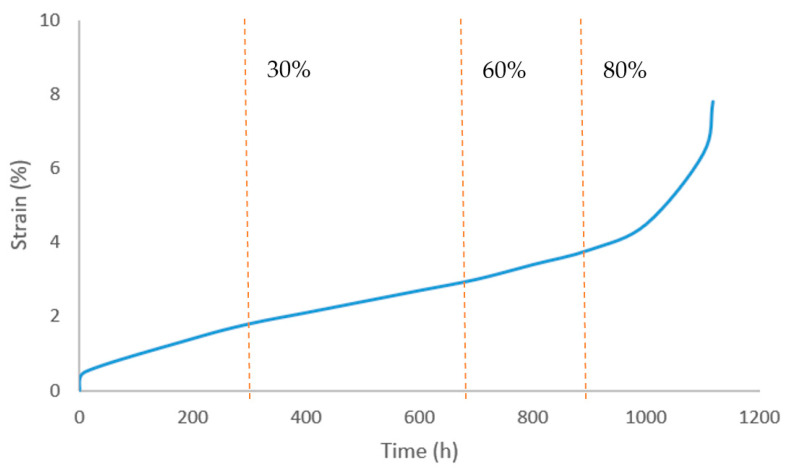
Creep curve of P92.

**Figure 5:**
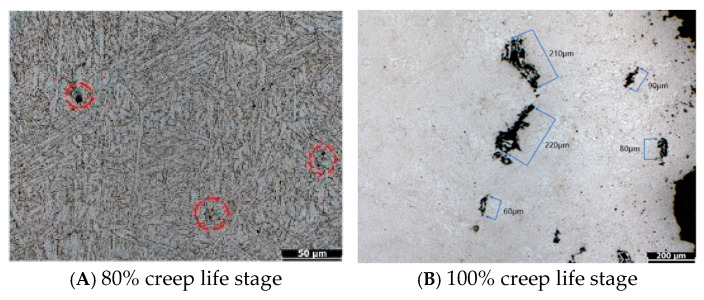
Optical microscope photos of specimens at 80% and 100% creep life stages.

To clarify the creep damage evolution law of P92 steel under 650 °C and 100 MPa working conditions, creep fracture and interruption experiments were conducted to obtain the creep curve of the entire life and the metallographic structure characteristics at different life stages. The creep fracture life of P92 steel specimen under this condition is 1117 h, and the creep curve shows a typical three-stage characteristic: the 0–30% life is the deceleration creep stage, and the strain growth rate gradually decreases; 30%~60% of the lifespan is in the steady-state creep stage, with a constant strain growth rate; and after 60% of its lifespan, it enters the accelerated creep stage, where the strain rapidly increases until it fractures. The creep curve of P92 is shown in Figure 4. The optical microscope photos of specimens at 80% and 100% creep life stages are shown in Figure 5.

Metallographic preparation and observation were conducted on samples at 80% and 100% life stages. The results showed that, in the 80% medium-to-high-damage stage, the number of grain boundary micropores significantly increases, and no connected microcracks are formed, with micrometer-level pores still being the main form of damage. During the 100% high-damage stage, micropores further grow and aggregate, and microcrack formation initiates at grain boundaries. The metallographic analysis results highly correspond to the changes in magnetoacoustic signal characteristics, providing a direct microstructural basis for the correlation between signal characteristics and creep damage.

### 4.2. Optimization and Determination of Magnetizing Parameters

The waveform, frequency, and current of the magnetization signal are the core parameters that affect the response strength of MBN and MAE signals. In this experiment, based on the magnetic characteristics of P92 steel and the performance of existing equipment, the optimal excitation parameters are determined to be 20 Hz and 0.3 A sine wave. The parameter selection is consistent with the widely recognized excitation parameters for high-temperature ferrite/martensitic steel magnetic detection in the field [2,11]. The low-frequency (≤20 Hz) alternating magnetic field can match the relaxation time of the P92 steel magnetic domain wall, achieving the sufficient irreversible jumping and rotation of the magnetic domain wall. The magnetic field strength (about 1.5 kA/m) generated by a 0.3 A excitation current exceeds the coercive force range of P92 steel, which ensures the efficiency of magnetic domain motion excitation while avoiding magnetic saturation caused by excessively high magnetic fields.

**Figure 6:**
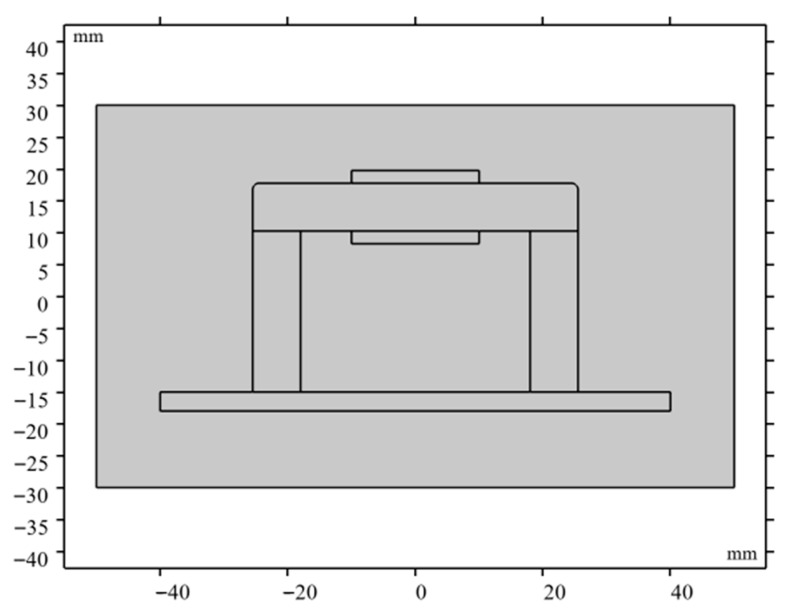
The magnetic circuit model.

**Figure 7:**
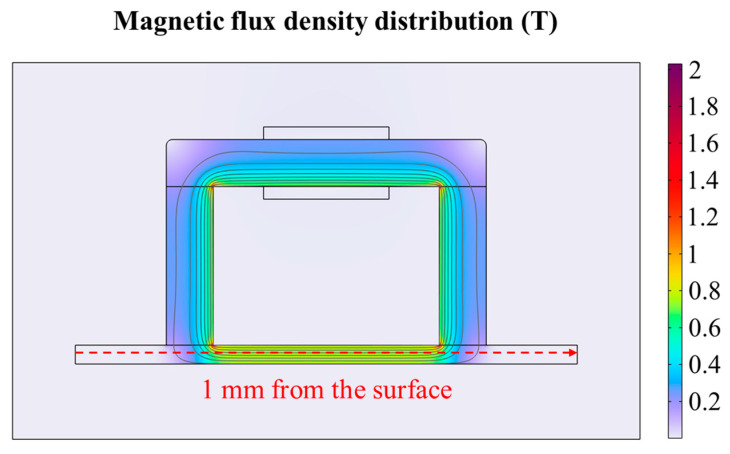
The magnetic flux density distribution.

**Figure 8:**
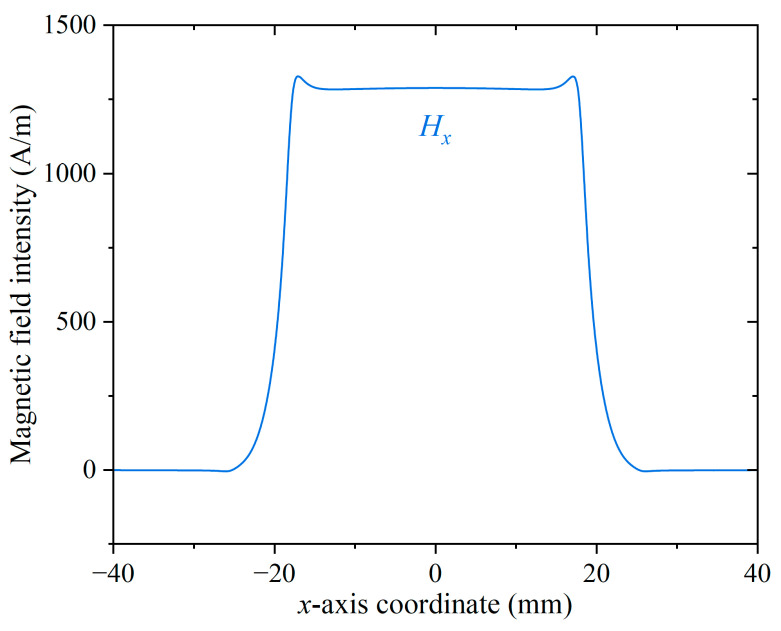
The magnetic field distribution of P92.

COMSOL Multiphysics 6.3 is used to simulate the magnetic field distribution of P92 specimen. The magnetic circuit model is shown in Figure 6. The yoke material is silicon steel NG0 35JN200. A free triangular mesh is adopted. The minimum element size is 0.002 mm. The magnetic flux density distribution can be shown in Figure 7. The magnetic field distribution of P92 is shown in Figure 8. From Figure 7 and Figure 8, it can be seen that the sample is uniformly magnetized.

In the experiment, sine wave is preferred as the magnetizing waveform, mainly based on the limitation of device compatibility. Waveforms with step features such as square waves and triangular waves require higher sampling rates and power amplifier bandwidths to ensure waveform integrity. However, the sampling rate (1 MHz) of ART USB2872 acquisition card and the bandwidth characteristics of TDA2030 amplifier are difficult to accurately reproduce in step signals, which can easily lead to waveform distortions. The continuous variation characteristics of sine waves are adapted to the performance parameters of existing equipment, which can stably output undistorted magnetizing signals, ensuring the continuous adjustability of magnetic field strength and consistency of MBN and MAE responses.

### 4.3. Collection and Filtering of MBN and MAE Signals

Using a 20 Hz, 0.3 A sine wave magnetizing parameter, the MBN and MAE signals of the P92 specimen in its initial state (without creep) was collected. The original signal was amplified by an SAEPA2 preamplifier (gain 40 dB) and transmitted to an ART USB2872 acquisition card (sampling rate of 1 MHz) for digital conversion. Then, it was processed using the Butterworth bandpass filter on the LabVIEW 2023 software. The MBN signal was filtered in the 10–30 kHz frequency band and the MAE signal was filtered in the 100–200 kHz frequency band to suppress environmental noise and power frequency interference.

**Figure 9:**
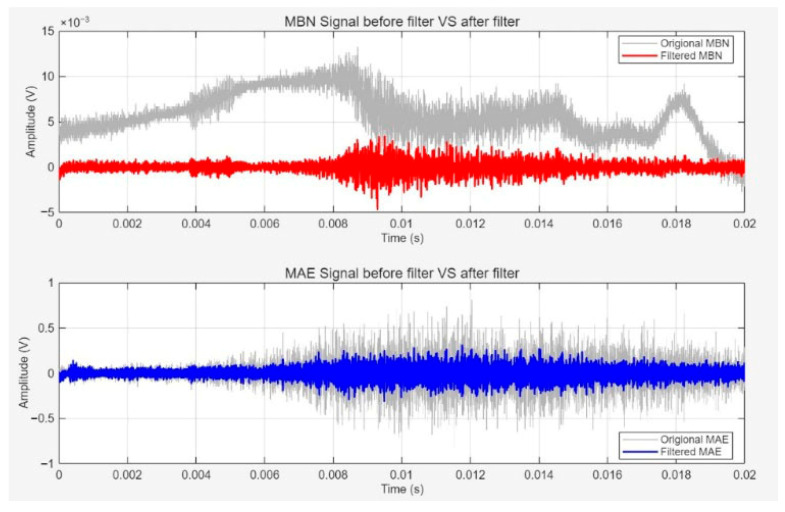
MBN and MAE signals before and after filtering.

Figure 9 shows the waveforms of MBN and MAE signals before and after filtering. The original MBN signal (gray curve) has obvious high-frequency noise with amplitude fluctuations of ±12 mV. After filtering (red curve), the noise is effectively suppressed and the signal baseline is stable within ±2 mV, while retaining the pulse characteristics of the MBN signal. The original MAE signal (light gray curve) also has broadband noise with amplitude fluctuations of ±0.8 V. After filtering (blue curve), the noise amplitude drops to within ±0.2 V and the elastic wave pulse characteristics of the signal are clearer. This preprocessing effect validates the rationality of the filtering parameters and provides a high-quality signal foundation for subsequent feature extraction.

**Figure 10:**
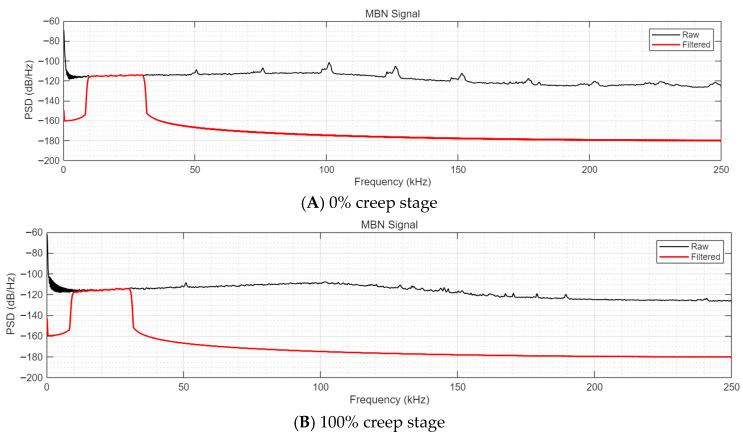
Power spectral density of raw and filtered MBN signals at 0% and 100% creep stages.

**Figure 11:**
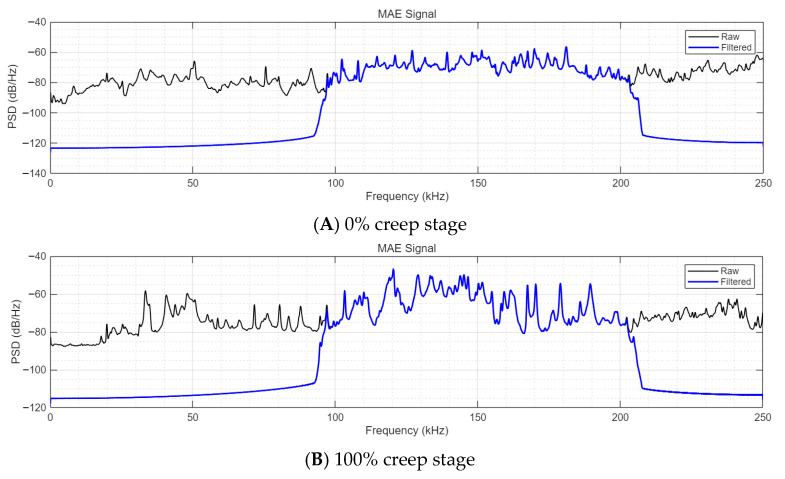
Power spectral density of raw and filtered MAE signals at 0% and 100% creep stages.

To further verify that the selected filtering bands retain all damage-related frequency components and exclude only invalid noise, power spectral density (PSD) analysis based on the Welch method was conducted on the raw and filtered MBN and MAE signals of P92 steel at 0% (uncrept) and 100% (complete creep) creep stages, with the results presented in Figure 10 and Figure 11. The PSD analysis parameters were consistent with the experimental signal acquisition settings: Hamming window with a length of 2048, overlapping points of 1024, 4096 FFT points, and a sampling frequency of 500 kHz. The filtering bands for MBN and MAE signals were set as 10~30 kHz and 100~200 kHz, respectively, consistent with the Butterworth bandpass filtering parameters in this study.

The PSD results of MBN signals (Figure 10) show that, for both 0% and 100% creep stages, the effective frequency components associated with magnetic domain wall motion and creep damage evolution are entirely concentrated in the 10~30 kHz band. The raw MBN signals exhibit significant low-frequency noise interference below 10 kHz and high-frequency noise interference above 30 kHz; after bandpass filtering, the invalid noise outside the target band is effectively suppressed, while all core spectral characteristics (e.g., peak frequency, energy distribution) of the raw signals related to the material’s magnetic properties and creep damage are completely retained in the 10~30 kHz band.

For the MAE signals (Figure 11), the effective elastic wave components induced by the magnetostrictive effect and creep damage are concentrated in the 100~200 kHz band for both creep stages. The original MAE signal has significant noise interference below 100 kHz and a poor correlation with creep. No effective creep characteristics were observed in the high-frequency range above 200 kHz. The 100~200 kHz bandpass filtering only removes the out-of-band noise, and the key spectral features reflecting the creep damage state of P92 steel are clearly preserved and highlighted in the filtered signals, with no loss of damage-related frequency components.

The above spectral density analysis directly validates the rationality and scientificity of the selected filtering bands for the MBN and MAE signals. The filtering process effectively suppresses the invalid noise that interferes with signal feature extraction, while fully retaining all frequency components associated with the intrinsic magnetic properties of P92 steel and the evolution of creep damage. This ensures that the subsequent extraction of time-domain and frequency-domain features is based on high-quality, damage-related signal components, laying a solid foundation for the accurate characterization of creep damage using MBN and MAE signals.

### 4.4. Analysis of Signal Characteristics at Different Creep Stages

Based on the different creep damage stage experiment data, the time-domain features (Time MeanAbs, Time Peak, Pulse_count) and frequency-domain features (bandwidth) of MBN and MAE signals were extracted and their trends with the proportion of creep life were analyzed.

#### 4.4.1. Mean Absolute Values at Different Creep Damage Stages

**Figure 12:**
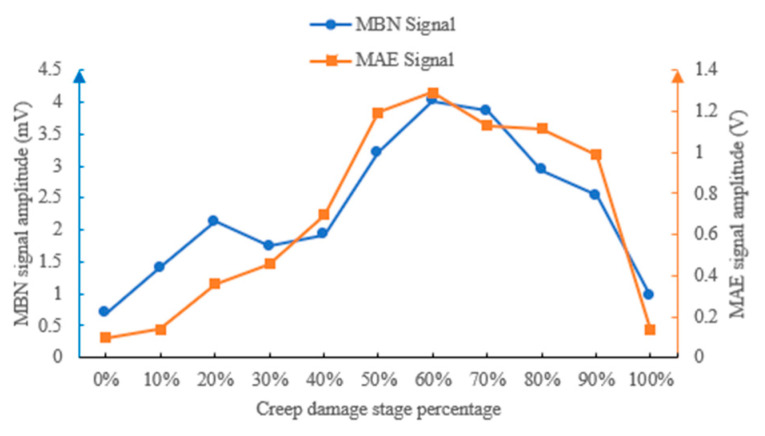
MBN and MAE mean absolute values at different creep stages.

Absolute mean reflects the overall energy accumulation level of the signal and shows a non-monotonic increasing trend with the increase in creep life proportion. The absolute mean variation trends of MBN and MAE signals at different creep stages are shown in Figure 12.

In the initial state (0% life), the absolute mean of MBN signal is 0.78 mV, and the MAE signal is 0.14 V. When the life proportion increases to 20%, the MBN signal increases to 0.95 mV and the MAE signal increases to 0.18 V, with a rate of increase of 21.8% and 28.6%, respectively. When the proportion of lifespan reaches 60%, the absolute mean of MBN signal surges to 4 mV and the MAE signal reaches 1.3 V, reaching their peak values. Then, as the degree of creep damage continues to increase, the absolute means of MBN and MAE signals decrease. This result is due to the continuous accumulation of micro defects during the creep process, i.e., an increase in dislocation density and an increase in the number of “pinning points” formed by Laves phase coarsening, which increases the resistance to magnetic domain wall movement and enhances the energy released by each magnetic domain jump, ultimately resulting in a continuous increase in the mean absolute value of the signal. The dispersed precipitates in the sample obviously increase, and the boundary of martensitic flat noodles gradually becomes unclear when the creep damage reaches 60% of the lifespan. The accumulated plastic deformation becomes large, severely restricting the flipping of magnetic domains during the magnetization process, resulting in a decrease in the absolute means of MBN and MAE signals with the increasing creep damage.

#### 4.4.2. Peak Values at Different Creep Damage Stages

The peak value reflects the maximum energy burst intensity of the signal, which shows a two-stage change of “rise first and then fall” with the increase in creep life proportion. In the 0%~60% life stage, the signal peak growth is gentle, i.e., the MBN signal increases from 4.68 mV to 14.18 mV, an increase of 202%. The MAE signal increased from 0.35 V to 4 V, with a 1043% increase. At this stage, the damage was mainly due to microstructural degradation, and no significant stress concentration was formed. After 60% lifespan, it enters a rapid leap stage. This is because, after 60% of the life of P92, the boundary of the martensite flat noodles gradually becomes unclear, which makes it more difficult for the magnetic domain wall jump to occur, and finally causes the signal peak to decrease after the lifetime exceeds 60%.

**Figure 13:**
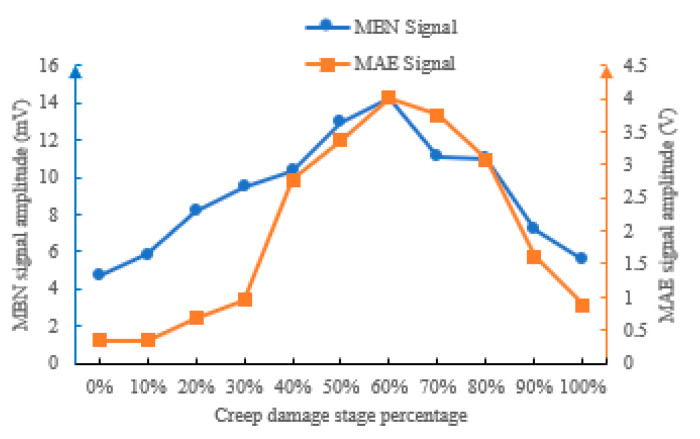
MBN and MAE peak values at different creep stages.

The peak value variation trends of MBN and MAE signals under different creep stages are shown in Figure 13.

#### 4.4.3. Pulse Counts at Different Creep Damage Stages

**Figure 14:**
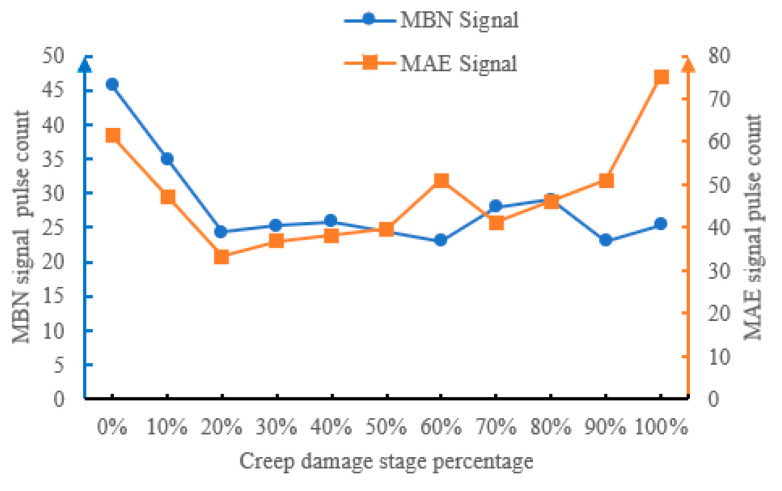
MBN and MAE pulse counts at different creep stages.

Pulse count reflects the number of triggering times of MBN and MAE responses per unit time, and shows three stages of surge–stable–slow with the increase in creep life proportion (Figure 14): In the 0%~20% life stage, the pulse count rapidly increases, which means pulse count is sensitive to early creep damage. During the 20% to 80% lifespan stage, the pulse count is basically stable, i.e., the MBN signal is maintained at 23–28 times/cycle, and the MAE signal is maintained at 33–46 times/cycle, which means pulse count is not sensitive to mid-term creep damage. During the 80% to 100% lifespan stage, the pulse count intensely fluctuates and increases. This is because micro defects are densely generated during this stage, and local magnetoacoustic responses are excited around each defect.

#### 4.4.4. Bandwidth at Different Creep Damage Stages

**Figure 15:**
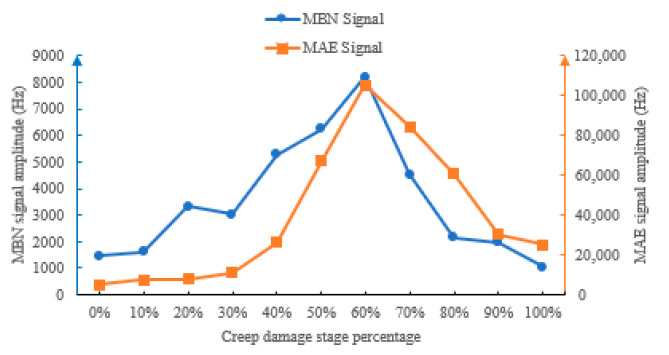
MBN and MAE bandwidths at different creep stages.

Bandwidth reflects the distribution range of signal frequency components, and shows a trend of “increase–maximum–decrease” with the increase in creep life proportion (Figure 15). In the 0%~30% and larger than 90% life stage, the signal bandwidth is basically stable in the narrowband range, i.e., the MBN signal bandwidth is maintained at 1–3 kHz, and the MAE signal bandwidth is maintained at 0.3–3 kHz, which means the internal structure of the material is uniform, and the frequency distribution of MBN and MAE responses are concentrated. After 30% lifespan, the bandwidth begins to expand and reach its maximum at 60% lifespan. Then, the bandwidths of MBN and MAE decrease rapidly. The essence of this phenomenon is the diversification of magnetoacoustic response mechanisms caused by creep damage. In the early stage, it is only a routine jump of magnetic domain walls, with frequency concentration. Due to complex damages such as microcracks and grain boundary separation in the mid-lifetime, the different frequencies of magnetoacoustic responses are excited, ultimately leading to a significant expansion of the signal bandwidth. In the later stage of creep damage, significant plastic deformation limits the free variation in magnetic domains, resulting in a smaller bandwidth.

### 4.5. PCA Creep Damage Evaluation Based on Feature Dimensionality Reduction and Damage Clustering Analysis of MBN and MAE Signals

The MBN and MAE signal parameters, which include absolute mean value, peak value, pulse count, and bandwidth, are all related to creep damage. Due to the eight parameters of time- and frequency-domain characteristic, and the non-monotonic correspondence between each characteristic parameter and the degree of creep damage, it is necessary to perform dimensionality reduction on the signal features and find feasible creep damage quantification methods. Principal component analysis (PCA) was conducted jointly on the full eight-dimensional combined feature matrix of MBN and MAE (four MBN features: absolute mean, peak value, pulse count, and bandwidth; four MAE features: absolute mean, peak value, pulse count, and bandwidth), in accordance with the standard PCA methodology. The eigenvalues, variance contribution rates and cumulative variance contribution rates of the extracted principal components are presented in Table 1, and the first two principal components (PC1 and PC2) with the highest cumulative variance contribution rate were selected to construct the two-dimensional principal component space for creep damage clustering analysis.

In the experiment, 18 sets of sample data are collected for each creep grade (0%, 10%, 20%, 30%, 40%, 50%, 60%, 70%, 80%, 90%, 100%). Based on the joint PCA results of the combined feature matrix, the PCA characteristic scatter plot of P92 steel creep grade is obtained as shown in Figure 14.

**Figure 16:**
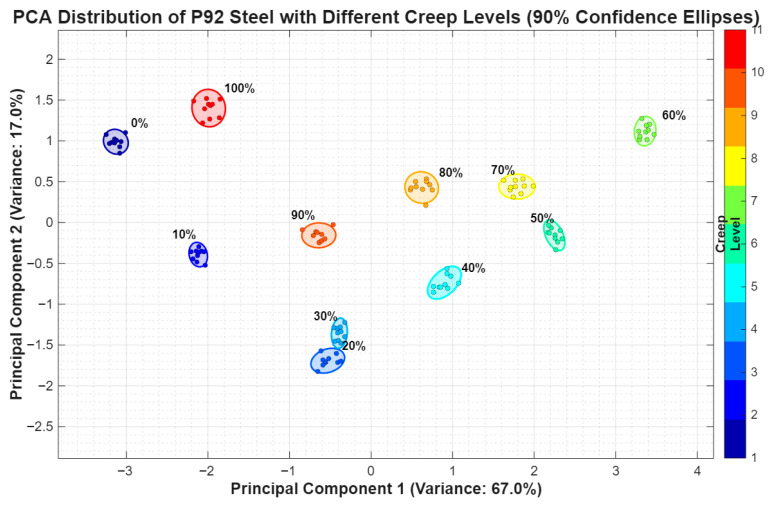
PCA distribution of P92 steel with different creep levels.

From Figure 16, it can be seen that samples with different proportions of creep life exhibit a gradient clustering distribution in the two-dimensional principal component space, and show a clear spatial migration pattern as the creep level increases. The samples with low creep levels (0%~20%) are concentrated in the lower left region of the feature space. The PC1 score interval is [−6, −1] and the PC2 score interval is [−4, −2]. The 90% confidence ellipses of each level do not overlap—the 0% life samples (red) are located on the leftmost side, the 10% life samples (orange) migrate to the right, and the 20% life samples (yellow) further approach the center. This distribution feature indicates that the damage level is relatively light at this stage, and the differences in signal characteristics have significant discriminability.

Samples with moderate creep levels (30%~60%) migrate to the middle-right region of the feature space, while the PC1 score interval expands to [1, 3] and the PC2 score interval rises to [0, 2]. A total of 30% of the samples (light yellow) are in the middle-left transition zone, while 40% and 60% of the samples in turn diffuse to the right. The area of the confidence ellipse slightly increases with the increase in creep levels, reflecting the continuous accumulation of micro defects at this stage, leading to a gradual increase in the discreteness of signal features.

The samples with high creep levels (70%~90%) are concentrated in the upper-right region of the feature space, with the PC1 score interval reaching [2, 4] and the PC2 score interval rising to [2, 4]. The confidence ellipses of 50%, 70%, 80% and 90% life samples shift to the lower side as the creep level increases, reflecting the gradual change in signal characteristics with defect evolution in the high-damage stage. At the same time, the color gradient (from bright yellow to deep blue) in Figure 9 is positively correlated with the creep level (0% to 100%), intuitively presenting the correspondence between the principal component space position and the degree of creep damage, indicating that the PCA reduced magnetoacoustic features from the combined feature matrix can effectively distinguish different creep stages except those of some of the 40% and 90% samples.

To transform the intuitive clustering trend in Figure 16 into an objective quantitative basis for determining creep levels, this study supplemented two quantitative indicators: inter-class distance and cross-validation accuracy. The key results were presented in a table to systematically verify the level discrimination ability of PCA features.

#### 4.5.1. Inter-Class Distance: Quantifying the Degree of Level Separation

The inter-class distance reflects the separation ability of different creep levels in the PCA space, and the specific values are shown in Table 2. According to this table, the average inter-class distance for all levels is 2.61, with the 0% (no damage) and 60% levels having the largest distance (6.50). This corresponds to the complete separation of the two types of samples in the principal component space in Figure 16, which can be preliminarily determined by the PC1 range (0% level is concentrated in PC1 ∈ [−3.3, −2.9], and 100% level is concentrated in PC1 ∈ [−2.2, −1.8]). The minimum inter-class distance appears at the 20% and 30% levels (0.36), corresponding to a slight overlap in the confidence ellipses of the two types of samples in the figure. A further subdivision is needed in conjunction with other indicators, but overall distinguishability is still maintained and complete confusion has not occurred.

#### 4.5.2. Cross-Validation: Ensuring the Reliability of the Judgment Method

To verify the generalization ability of the creep level recognition method constructed by PCA features, this study used 5-fold cross-validation for evaluation.

The accuracy of 5-fold cross-validation reached 92%, with a classification error rate of 0.6%, indicating that the creep level recognition method based on MBN-MAE combined with PCA features has demonstrated perfect performance on the current dataset. This result not only proves the effectiveness of the method on experimental data, but also greatly enhances its confidence in promoting it to the same type of P92 steel creep damage detection scenario, providing extremely reliable technical support for the online determination of creep level of actual high-temperature components.

## 5. Conclusions

In this study, a method for analyzing and quantifying the creep damage of P92 steel based on MBN and MAE testing. The innovations of this work can be summarized in three key points:

(1) Successfully developed MAE-MBN magnetoacoustic composite measurement system

Based on the magnetic characteristics of P92 steel, a customized MAE-MBN joint measurement system is built. A U-shaped silicon steel magnetic yoke and a 600-turn magnetizing coil are used to construct a low-frequency alternating magnetic field excitation unit, which is adapted to 20 Hz, 0.3 A sine wave excitation parameters. Signals of MBN and MAE sensors are triggered by magnetizing signals to achieve synchronous acquisition. The SAEPA2 preamplifier and ART acquisition card are used for complete signal enhancement and digitization. The upper computer software achieves the integrated functions of excitation control, signal filtering, and data storage. The stability meets the requirements of long-term creep experiments.

(2) The correlation between the characteristics of MBN, MAE and creep damage of P92 steel is clarified

By extracting the characteristics of MAE and MBN signals under different creep life stages, the corresponding relationship between magnetoacoustic features and damage evolution is clarified. The time-domain features (absolute mean, peak value, pulse count) and frequency-domain features exhibit non-monotonic changes with the degree of creep damage. The time-domain and frequency-domain characteristics of MBN and MAE signals show significant non-monotonic inflection points at 60% creep life, which correspond to the height of the starting point of the acceleration stage of P92 steel creep. This signal inflection point can be used as the core signal feature for the mid-term warning of P92 steel creep damage. The signal variation patterns in the early stage (0%~60%) and later stage (60%~100%) of creep are caused by the combined effects of various micro factors such as dislocation proliferation, coarsening of precipitation phases, initiation and aggregation of micropores, and intensified plastic deformation. The early stage is dominated by dislocation proliferation and coarsening of Laves phases, while the later stage is dominated by the initiation and aggregation of micropores and intensified plastic deformation. The micro evolution mechanism has been verified by metallographic experiments and confirmed by the existing research literature on the creep of P92 steel [1,18].

It is difficult to determine the stage of creep damage solely based on one or a few characteristics of MBN or MAE.

(3) Effective clustering and grading of non-monotonic MBN and MAE features based on PCA

Considering the non-monotonic fluctuation in MBN and MAE features with the creep process, PCA is introduced to reduce the dimensionality of multidimensional MBN and MAE features. After mapping the non-monotonic original signal features to a two-dimensional principal component space, samples with different creep levels are exhibited in a clear gradient clustering distribution. The inter-class distances of the sample set in the low-, medium- and high-creep stages are significantly greater than the intra-class dispersion. The clustering boundary is highly consistent with the damage evolution law. This method solves the problem of difficulty in directly distinguishing the damage stage of non-monotonic features and achieves the visualization and accurate differentiation of the creep grade of P92 steel.

In summary, the MAE-MBN composite measurement system developed in this article provides a new technical means for detecting creep damage in P92 steel, while the PCA method effectively clusters non-monotonic MBN and MAE features, providing a feasible analysis path for identifying the in-service creep grade of P92 steel components in power plants. In the future, machine learning algorithms can be further combined to construct an intelligent creep damage recognition model based on MBN and MAE PCA features, which will enhance the practicality of the system further.

## Figures and Tables

**Table 1:** Eigenvalues and variance contribution rates of principal components for MBN-MAE combined features.

Principal Component	Eigenvalue	Variance Contribution Rate (%)
PC1	4.21	67
PC2	2.08	17.00
PC3	0.95	16

**Table 2:** Inter-class Euclidean distance matrix for different creep grades.

	0%	10%	20%	30%	40%	50%	60%	70%	80%	90%	100%
**0%**	0.00	1.80	3.82	3.62	4.39	5.53	6.50	5.02	3.85	2.86	1.26
**10%**	1.80	0.00	1.20	2.06	3.04	4.40	5.70	4.08	2.98	1.51	1.81
**20%**	3.82	1.20	0.00	0.36	1.80	3.22	5.00	3.11	2.37	1.50	3.53
**30%**	3.62	2.06	0.36	0.00	1.39	2.73	4.38	2.47	1.97	1.33	2.72
**40%**	4.39	3.04	1.80	1.39	0.00	1.52	2.50	1.49	1.64	1.62	3.56
**50%**	5.53	4.40	3.22	2.73	1.52	0.00	1.86	0.64	4.20	5.36	4.52
**60%**	6.50	5.70	5.00	4.38	2.50	1.86	0.00	1.17	1.71	4.27	5.30
**70%**	5.02	4.08	3.11	2.47	1.49	0.64	1.17	0.00	1.20	2.57	3.93
**80%**	3.85	2.98	2.37	1.97	1.64	1.71	2.50	1.20	0.00	1.30	2.79
**90%**	2.86	1.51	1.50	1.33	1.62	5.36	4.27	2.57	1.30	0.00	2.20
**100%**	1.26	1.81	3.53	2.72	3.56	4.52	5.30	3.93	2.79	2.20	0

## Data Availability

The original contributions presented in this study are included in the article. Further inquiries can be directed to the corresponding author.
